# Factors Associated With Remote Diffusion-Weighted Imaging Lesions in Spontaneous Intracerebral Hemorrhage

**DOI:** 10.3389/fneur.2018.00209

**Published:** 2018-04-06

**Authors:** Xiang-hua Ye, Ting Gao, Xu-hua Xu, Jin-song Cai, Jia-wen Li, Kai-ming Liu, Shui-jiang Song, Xin-zhen Yin, Lu-sha Tong, Feng Gao

**Affiliations:** ^1^Department of Neurology, The Second Affiliated Hospital, School of Medicine, Zhejiang University, Hangzhou, China; ^2^Department of Neurology, The Fourth Affiliated Hospital, School of Medicine, Zhejiang University, Yiwu, China; ^3^Department of Radiology, The Second Affiliated Hospital, School of Medicine, Zhejiang University, Hangzhou, China

**Keywords:** intracerebral hemorrhage, remote DWI lesions, glucose, white matter hyperintensity, small vessel disease

## Abstract

**Background and purpose:**

Remote diffusion-weighted imaging lesions (R-DWILs) have been detected in patients with spontaneous intracerebral hemorrhage (ICH) and may be correlated with clinical outcome. However, the mechanisms and characteristics of R-DWILs have not been fully elucidated. In this study, we sought to demonstrate the clinical characteristics of R-DWILs in spontaneous ICH.

**Methods:**

We prospectively collected data with spontaneous ICH patients from November 2016 to December 2017. In these patients, cerebral magnetic resonance imaging was performed within 28 days after ICH onset. R-DWIL was defined as a hyperintensity signal in diffusion-weighted imaging with corresponding hypointensity in apparent diffusion coefficient, and at least 20 mm apart from the hematoma. We compared two groups of patients with or without R-DWIL with the demographic and clinical characteristics, laboratory parameters, and imaging characteristics, by using univariate and multivariate analysis.

**Results:**

Of the 222 patients enrolled, a total of 75 R-DWILs were observed in 41 patients (18.5%). Among these lesions, the cortical and subcortical areas were the predominant locations with a proportion of 77.3%. The median diameter of R-DWILs was 7 mm (range 2–20 mm). Twelve patients were found having more than one lesion, with five among which showed R-DWILs in multiple cerebral arterial territories. In multivariate modeling, higher fasting glucose (OR 1.231; 95% CI 1.035–1.465; *p* = 0.019) and more severe white matter hyperintensity (WMH) (OR 6.589; 95% CI 2.975–14.592; *p* < 0.001) were independent factors related to the presence of R-DWILs.

**Conclusion:**

In our study, approximately one-fifth of ICH patients showed coexistence of R-DWIL. Higher fasting glucose and more severe WMH were associated with R-DWIL occurrence in spontaneous ICH.

## Introduction

Intracerebral hemorrhage (ICH) is a devastating condition with high morbidity and mortality (1). The recent application of magnetic resonance imaging (MRI) in ICH patients demonstrated a varied proportion (11.1–41.0%) of patients presented diffusion-weighted imaging (DWI) lesions remote from hematomas (2–11). The remote DWI lesions (R-DWILs) were typically small, cortical or subcortical, mostly subclinical and were found may correlated with poor prognosis of ICH patients (6, 11, 12). So far, the formation mechanisms and related risk factors of R-DWIL formation have rarely been studied. The aim of our current study was to illustrate the frequency and pattern of R-DWIL occurred with spontaneous ICH and try to identify related factors associated with R-DWIL. We hoped to present new aspects of ICH-related cerebral injury and add more information for its future therapy.

## Materials and Methods

### Study Population

We prospectively collected data on consecutively enrolled patients with the diagnosis of spontaneous ICH admitted to the stroke center of our hospital from November 2016 to December 2017. Spontaneous ICH patients would be considered eligible for our study when they had received baseline computed tomography (CT) scan on admission and clinical data, such as detailed and reliable history, as well as brain MRI consist of DWI, apparent diffusion coefficient (ADC), and fluid-attenuated inversion recovery (FLAIR) sequences within 28 days after ICH onset. Additional imaging tests, including angiography, enhanced-MRI, and MR or CT angiography, were applied if necessary to exclude secondary ICH cause such as aneurysm, vascular malformation, moyamoya disease, cavernous hemangioma, cerebral venous thrombosis, neoplasm, or hemorrhagic conversion of ischemic infarction. Patients only presented isolated intraventricular hemorrhage were not included. Those who had diagnosed hematologic abnormality or a head trauma history were also ruled out.

The study received ethical approval of the institutional Human Research Ethics Committee of the Second Affiliated Hospital of Zhejiang University.

### Imaging Acquisition

Computed tomography scans were performed using multidetector row scanners [Optima CT540, General Electric (GE) Healthcare, CT, USA; or SOMATOM Sensation 16, Siemens, German] with the following parameters: slice thickness 5 mm, 120 Kv, and 100–300 mAs. MRI was performed on 1.5-T (Sonata, Siemens, German) or 3.0-T scanner (Signa HDxt, GE Healthcare, CT, USA) with standardized protocol consisted of axial T1-weighted, T2-weighted, T2 FLAIR, DWI, and ADC sequences. Axial DWI sequences were acquired on 1.5T [repetition time (TR) 3,100 ms, echo time (TE) 84 ms, *b* = 0/1,000 s/mm^2^, 6-mm slice thickness, 0.5-mm gap, FOV 230 mm] or 3.0T scanner (TR 5,200 ms, TE 75 ms, *b* = 0/1,000 s/mm^2^, 6-mm slice thickness, 0.5-mm gap, FOV 240 mm) with different parameters.

### Data Collection

Baseline demographic (age, sex) characteristics have been recorded. Related medical situations, such as hypertension, diabetes, cardiac diseases related with cardioembolic infarction [i.e., atrial fibrillation (AF), mechanical prosthetic valve, recent myocardial infarction, dilated cardiomyopathy, infective endocarditis, and so forth] were evaluated. Smoking, alcohol and prior stroke, usage of antiplatelet or anticoagulant drugs, admission systolic blood pressure (SBP), time to take the MRI, Glasgow Coma Scale (GCS), and National Institute of Health Stroke Scale on admission, were obtained. Hypertensive and diabetic was confirmed if the patient had a documented medical history and had been treated with medication. Smoking and alcohol assumption included previous and current situations. Laboratory parameters [platelet count, hemoglobin, homocysteine, cholesterol, low-density lipoprotein cholesterol, C-reactive protein (CRP), creatinine, fasting glucose, fibrinogen, prothrombin time (PT), activated partial thromboplastin time (APTT)] were acquired the next morning after admission. Imaging characteristics [location and volume of the hematoma, presence of ventricular or subarachnoid extension, white matter hyperintensity (WMH) severity, presence and characteristics of R-DWIL] were collected. A stroke physician (Lu-sha Tong) and a radiologist (Jin-song Cai) read the data of R-DWIL independently and reached consensus (Kappa = 1.0). The evaluation of WMH severity was performed by one experienced reader (Xiang-hua Ye) who was blind to the DWI study.

### Assessment of Hematoma

Initial CT scan was applied for determining the location and volume of hematoma. ICH locations were categorized as lobe, deep structure, brainstem, or cerebellum. Deep structures consist of basal ganglia, thalamus, and internal capsule. Hematoma volume was calculated using the ABC/2 method (13). The presence of ventricular or subarachnoid extension was also recorded.

### Assessment of WMH

White matter hyperintensity was defined as signal abnormality in white matter, which usually demonstrated hypointensity in T1-weighted image, and hyperintensity in T2-weighted and FLAIR image. Periventricular white matter hyperintensity (PV-WMH) and deep white matter hyperintensity (D-WMH) were assessed, respectively, according to Fazekas scale (14), with scores from 0 to 3. The overall severity of WMH was calculated as the total of PV-WMH and D-WMH scores. A total score greater than 2 was defined as high-grade WMH in this study.

### Assessment of R-DWIL

**Figure 1:**
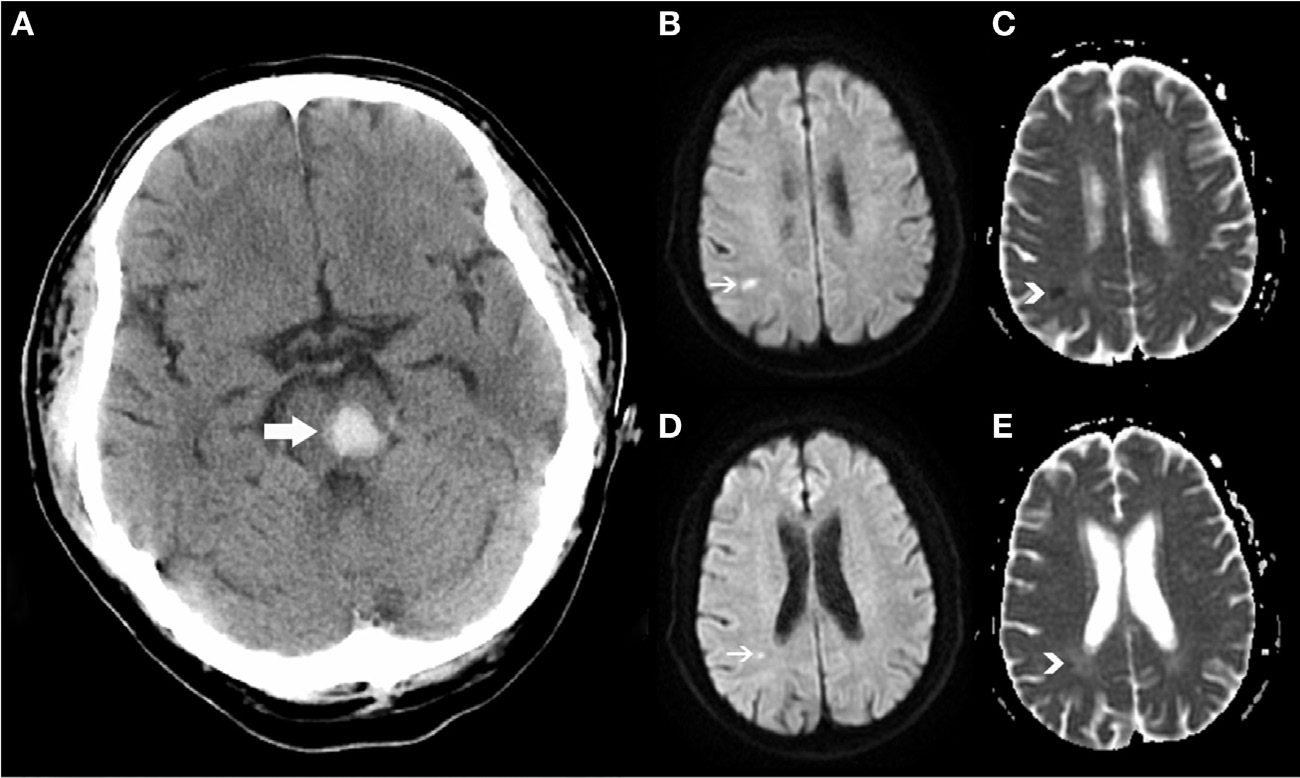
Positive remote diffusion-weighted imaging lesions in a 54-year-old man with left pontine hemorrhage **(A)**. Diffusion-weighted imaging shows hyperintensity lesions **(B,D)** on subcortical areas of parietal and occipital lobes, with corresponding low signal intensity in apparent diffusion coefficient map **(C,E)**.

Remote DWI lesion was defined as hyperintensity lesion in DWI, measuring less than 20 mm in diameter, with corresponding low signal intensity in ADC map (Figure 1). Restricted diffusion within or adjacent to the hematoma (<20 mm) was excluded from our analysis. DWI sequence and ADC map were read coherently to identify the R-DWILs, and characterize their sizes and locations if present. Their sizes were represented by the maximum diameters calculated in DWI scans, and their locations were divided into lobe, deep structure, brainstem, and cerebellum. We recorded their laterality relative to hematomas, which were defined as ipsilateral, contralateral, or unclassified (especially in the case of brainstem hemorrhage involving both sides). Among patients with more than one R-DWIL, we further evaluated if the lesions were in multiple arterial territories (lesions involving bilateral anterior cerebral circulation, or both anterior and posterior circulation).

### Statistical Analysis

We compared the demographic and clinical characteristics, laboratory parameters, and imaging characteristics between patients with or without R-DWIL. Continuous variables are presented as mean (SD) or median [interquartile range (IQR)] according to whether the variables are normal distribution, and categorical variables are presented as number (percentage). Univariate analyses were conducted by using 2-sample *t* test or Mann–Whitney *U* test for continuous variables and χ^2^ test or Fisher exact test for categorical variables, as appropriate. Variables with probability value <0.1 on univariate analyses were allowed for multivariate logistic regression modeling, with some noted exceptions. A *p* value ≤ 0.05 was considered statistically significant. All statistical analyses were performed using IBM SPSS Statistics version 23.0.

## Results

Of 300 spontaneous ICH patients admitted to our center, 78 were excluded for the following reasons: (1) isolated intraventricular hemorrhage (*n* = 4); (2) secondary ICH due to aneurysm (*n* = 1), vascular malformation (*n* = 6), moyamoya disease (*n* = 2), cavernous hemangioma (*n* = 5), neoplasm (*n* = 2); and (3) without or with low-quality imaging sans (*n* = 58). Finally, 222 patients were included in further analysis, among whom the mean age was 59.9 ± 13.4, and 133 (59.9%) were male. One hundred and sixty-two (73.0%) patients had previously diagnosed hypertension and 36 (16.3%) had a history of cerebral vascular events, including ICH and ischemic stroke/transient ischemic attack (15). The past cardiac diseases histories were obtained and routine tests were run including 24-h Holter monitoring and echo cardiogram during the hospitalization, and the percentage of AF in two patients group was comparable. The enrolled patients had overall high degree of consciousness and 78.8% of their GCS were 15. The median of days from ICH onset to MRI was 6. About 93.7% patients performed MRI within 14 days after ICH onset, among whom 73.6% within 7 days.

### Distribution and Size of Hematoma

The most common hematoma location was deep structures (148; 66.7%), followed by the lobe (42; 18.9%), brainstem (15; 6.8%), and cerebellum (10; 4.5%), with 7 (3.2%) patients had multiple locations of hematomas. The median volume of hematomas was 8.7 ml. The total frequency of patients with bleeding extending into ventricle or subarachnoid space was 38.3%.

### Presence and Characteristics of R-DWIL

**Figure 2:**
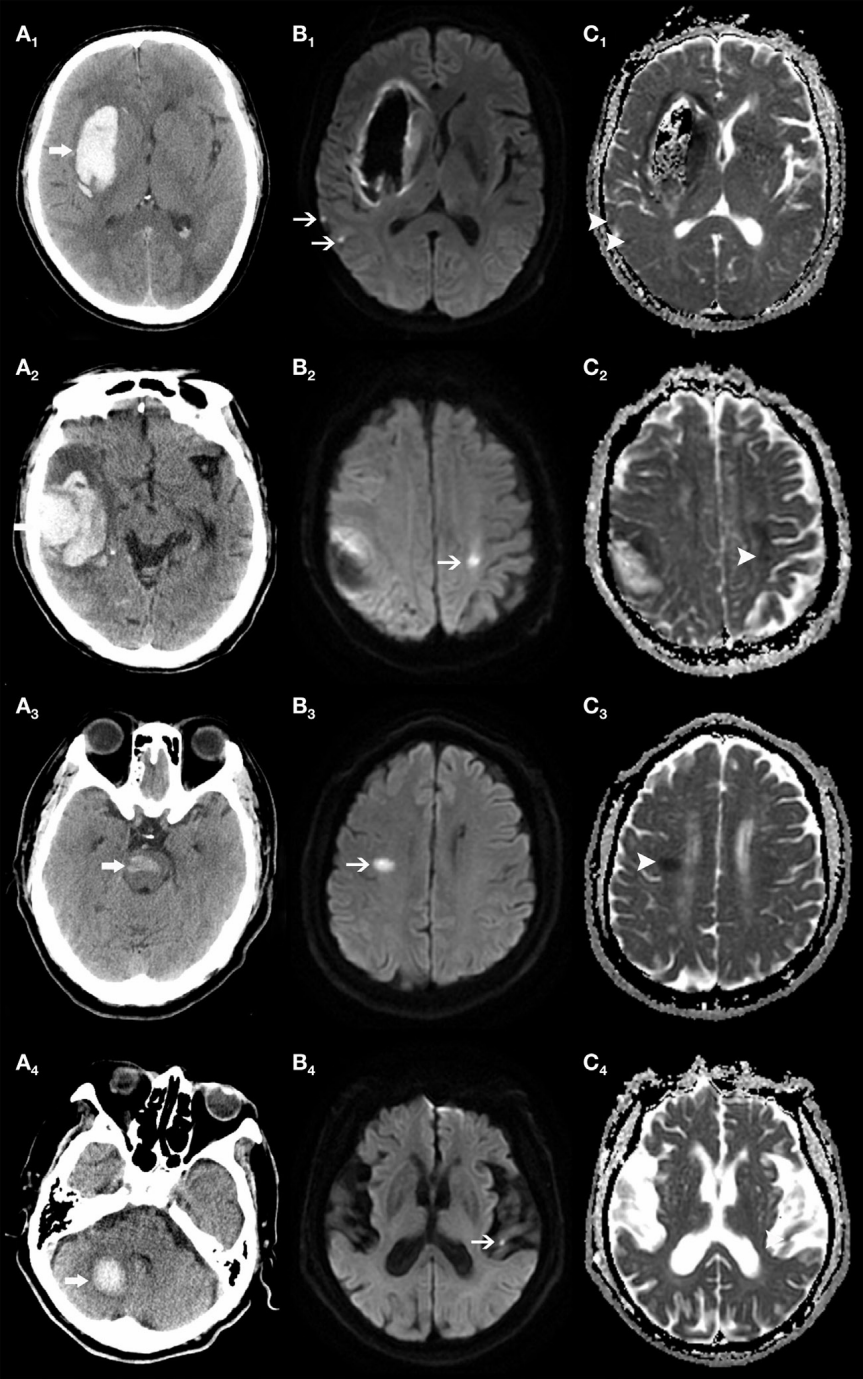
Remote DWI lesions (R-DWILs) are present in patients with bleeding at various sites. The computed tomography shows four patients with intracerebral hemorrhage in right basal ganglia (A_1_), right temporal lobe (A_2_), brainstem (A_3_), and right cerebellum (A_4_), with R-DWILs located at ipsilateral temporal cortex (B_1_, C_1_), contralateral postcentral gyrus (B_2_, C_2_), right centrum semiovale of frontal lobe (B_3_, C_3_), and contralateral temporal cortex (B_4_, C_4_) in diffusion-weighted imaging and ADC, respectively.

Forty-one (18.5%) of enrolled patients had one or more R-DWILs (median 1, range 1–8), no matter where the parenchymal hemorrhage originated, such as the deep structure, lobe, brainstem, and cerebellum (Figure 2). The total number of R-DWILs was 75, and none of them lead to significant clinical symptoms. All lesions were at least 20 mm away from the closest hematoma margin; their median diameter was 7 mm (range 2–20 mm). As shown in Table 1, 77.3% of the lesions located at cortical or subcortical areas of lobes (occipital lobe 30.7%; parietal lobe 16.0%; frontal lobe 16.0%; temporal lobe 8.0%; occipito-parietal area 6.7%); 14.7% at deep structures; 5.3% at brainstem; and 2.7% at cerebellum. In terms of laterality, 48% lesions were ipsilateral to the hematoma, whereas 45.3% contralateral and 6.7% unclassified. Twelve patients were found with multiple lesions, while five of them had lesions distributing in varied arterial territories.

**Table 1:** Distributions and proportions of remote DWI lesions.

Lobe					Deep	Brainstem	Cerebellum
Cortical: 16 (21.3%); subcortical: 42 (56%)				
Frontal	Temporal	Parietal	Occipital	Occipito-parietal	11 (14.7%)	4 (5.3%)	2 (2.7%)
12 (16.0%)	6 (8.0%)	12 (16.0%)	23 (30.7%)	5 (6.7%)			

Tables 2–4 summarize the baseline demographic and clinical characteristics, laboratory parameters, and imaging characteristics of the whole cohort, and the subgroups with “positive” and “negative” R-DWILs. All variables with *p* value < 0.1 in univariate analysis entered into multivariate logistic regression analyses, with exception of D-WMH and PV-WMH score, due to their strong correlation (*p* < 0.001) with each other as well as with high-grade WMH. Furthermore, factor of “fibrinogen” was not included into multivarivate analyses. Since it has close relation with other coagulation index, such as PT and APTT, which showed no statistical correlation with R-DWIL. Moreover, “fibrinogen” is a comprehensive and vulnerable parameter that responding to various events, such as systemic inflammation, tissue injury, and so on. It was thought to be elevated as CRP did. Ultimately, age, admission SBP, CRP, fasting glucose, ventricular extension, previous ischemic stroke/TIA, and high-grade WMH were adopted into multivariate modeling. As demonstrated in Table 5, factors independently associated with the positive R-DWIL were higher fasting glucose (OR 1.231; 95% CI 1.035–1.465; *p* = 0.019) and high-grade WMH (OR 6.589; 95% CI 2.975–14.592; *p* < 0.001).

**Table 2:** Demographic and clinical characteristics of patients with and without R-DWIL.

Characteristics	All subjects (*n* = 222)	R-DWIL negative (*n* = 181)	R-DWIL positive (*n* = 41)	*p-*Value
**Demographics**				
Age, years, mean (SD)	59.9 (13.4)	59.0 (13.1)	64.1 (13.8)	0.028
Male, *n* (%)	133 (59.9)	107 (59.1)	26 (63.4)	0.612
**Clinical**				
Hypertension, *n* (%)	162 (73.0)	131 (72.4)	31 (75.6)	0.674
Diabetes, *n* (%)	18 (8.1)	13 (7.2)	5 (12.2)	0.456
Previous ICH, *n* (%)	21 (9.5)	17 (9.4)	4 (9.8)	0.936
Previous ischemic stroke/TIA, *n* (%)	15 (6.8)	9 (5.0)	6 (14.6)	0.060
Atrial fibrillation, *n* (%)	4 (1.8)	2 (1.1)	2 (4.9)	0.322
Antiplatelet use, *n* (%)	10 (4.5)	8 (4.4)	2 (4.9)	1.000
Anticoagulant use, *n* (%)	3 (1.4)	3 (1.7)	0 (0.0)	0.935
Smoking, *n* (%)	64 (28.8)	49 (27.1)	15 (36.6)	0.225
Alcohol, *n* (%)	51 (23.0)	42 (23.2)	9 (22.0)	0.863
Admission SBP, mmHg, median (IQR)	156.5 (27.0)	155.0 (25.5)	165.0 (34.5)	0.004
Time to MRI, day, median (IQR)	6 (4)	6 (4)	6 (5)	0.577
Baseline GCS, median (IQR)	15 (0.0)	15 (0.0)	15 (0.5)	0.512
Baseline NIHSS, median (IQR)	5 (9.0)	5 (9.0)	5 (8.5)	0.717

*R-DWIL, remote diffusion-weighted imaging lesion; ICH, intracerebral hemorrhage; TIA, transient ischemic attack; SBP, systolic blood pressure; IQR, interquartile range; MRI, magnetic resonance imaging; GCS, Glasgow Coma Scale; NIHSS, National Institute of Health Stroke Scale*.

**Table 3:** Laboratory parameters of patients with and without R-DWIL.

Parameters	All subjects (*n* = 222)	R-DWIL negative (*n* = 181)	R-DWIL positive (*n* = 41)	*p-*Value
Platelet, ×10^9^/l, mean (SD)	202.2 (67.4)	203.0 (68.0)	199.0 (65.1)	0.733
Hemoglobin, g/l, median (IQR)	138 (26.3)	137 (26.5)	139 (25.5)	0.749
Homocysteine, μmol/l, median (IQR)	13.9 (6.2)	13.8 (6.5)	15.8 (6.0)	0.186
Cholesterol, mmol/l, mean (SD)	4.8 (1.0)	4.8 (1.0)	4.9 (1.0)	0.534
LDL-C, mmol/l, mean (SD)	2.6 (0.8)	2.6 (0.8)	2.7 (0.7)	0.623
CRP, mg/l, median (IQR)	5.2 (9.2)	4.7 (7.4)	7.5 (20.5)	0.082
Creatinine, μmol/l, median (IQR)	59 (23.3)	59 (23.5)	59 (27.5)	0.403
Fasting glucose, mmol/l, median (IQR)	5.6 (1.7)	5.5 (1.4)	6.6 (2.0)	< 0.001
Fibrinogen, g/l, median (IQR)	3.4 (1.2)	3.4 (1.3)	3.7 (1.7)	0.039
PT, s, median (IQR)	13.2 (1.0)	13.2 (1.0)	13.2 (1.1)	0.525
APTT, s, median (IQR)	36.2 (6.3)	36.2 (5.5)	34.4 (7.5)	0.195

*R-DWIL, remote diffusion-weighted imaging lesion; IQR, interquartile range; LDL-C, low-density lipoprotein-cholesterol; CRP, C-reactive protein; PT, prothrombin time; APTT, activated partial thromboplastin time*.

**Table 4:** Imaging characteristics of patients with and without R-DWIL.

Characteristics	All subjects (*n* = 222)	R-DWIL negative (*n* = 181)	R-DWIL positive (*n* = 41)	*p*-Value
Hematoma volume, ml, median (IQR)	8.7 (15.7)	8.5 (15.4)	9.7 (19.7)	0.494
ICH location, *n* (%)				0.708
Lobe	42 (18.9)	35 (19.3)	7 (17.1)	
Deep structure	148 (66.7)	123 (68.0)	25 (61.0)	
Brainstem	15 (6.8)	11 (6.1)	4 (9.8)	
Cerebellum	10 (4.5)	7 (3.9)	3 (7.3)	
Multiple locations	7 (3.2)	5 (2.8)	2 (4.9)	
Ventricular extension, *n* (%)	66 (29.7)	46 (25.4)	20 (48.8)	0.003
Subarachnoid extension, *n* (%)	19 (8.6)	15 (8.3)	4 (9.8)	0.945
WMH Fazekas score				
PV-WM, *n* (%)				0.001
0	42 (18.9)	40 (22.1)	2 (4.9)	
1	101 (45.5)	87 (48.1)	14 (34.1)	
2	43 (19.4)	31 (17.1)	12 (29.3)	
3	36 (16.2)	23 (12.7)	13 (31.7)	
D-WM, *n* (%)				<0.001
0	73 (32.9)	67 (37.0)	6 (14.6)	
1	74 (33.3)	68 (37.6)	6 (14.6)	
2	40 (18.0)	26 (14.4)	14 (34.1)	
3	35 (15.8)	20 (11.0)	15 (36.6)	
High grade WMH (>2), *n* (%)	88 (39.6)	57 (31.5)	31 (75.6)	<0.001

*R-DWIL, remote diffusion-weighted imaging lesion; IQR, interquartile range; ICH, intracerebral hemorrhage; WMH, white matter hyperintensity; PV-WM, periventricular white matter; D-WM, deep white matter*.

**Table 5:** Multivariate logistic regression analyses for presence of R-DWIL.

Variables	OR	95% CI	*p-*Value
Fasting glucose	1.231	1.035–1.465	0.019
High-grade WMH	6.589	2.975–14.592	<0.001

*R-DWIL, remote diffusion-weighted imaging lesion; OR, odds ratio; CI, confidence interval; WMH, white matter hyperintensity*.

## Discussion

The present study found that R-DWILs occurred in a proportion of spontaneous ICH patients in our center. They were usually small and predominately located in cortical or subcortical areas of lobes. None of these patients had presented related clinical symptoms.

Our study provided new insights into topography of R-DWILs. We found that the occipital and occipital-parietal region showed the highest percentage of R-DWIL occurrence. Some previous studies referring to reversible posterior leukoencephalopathy syndrome have shown that the posterior cerebral region was susceptible to abruptly changed BP, altered endocrine status, and some other injuries, thus leading to cerebral structural damages (16, 17).

Among patients with more than one R-DWIL, some showed lesions in multiple cerebral arterial territories, which may implicate a source of cardiac embolism. However, we hardly found association between the risk factors of cardiac embolism and R-DWIL presentation. On the other hand, cardiac embolism often lodges in distal arteries supplying the cerebral cortex while small vessel occlusion affects subcortical tissue (18). In our study, the percentage of lesions in subcortical area far exceeded that of cortex. There is another possibility of R-DWIL formation as *in situ* thrombolism due to small vessel obstructions. Our work had demonstrated more extensive high grade WMH in ICH patients with positive R-DWIL compared to their negative counterparts. Piles of studies had reported the positive correlation between cerebral small vessel disease (CSVD) and R-DWIL presence after primary ICH (5, 6, 8, 12). WMH is a maker of CSVD, currently described as diffuse, intrinsic disease of the smaller (40–200 µm diameter) arterioles, referred by Fisher et al. as arteriolosclerosis, lipohyalinosis, or fibrinoid necrosis (19). Recent studies have suggested that in acute ICH patients, the dynamic cerebral autoregulation (dCA) was globally impaired in about 2 weeks, which we suppose, may serve as the cause of small vessel obstructions (20, 21). The authors also suggested that a pre-existed dysfunction of dCA before ICH exists. In this context, R-DWIL may be due to the small vessel dysregulation and the pre-existed CSVD burden may somehow indicate the potential risk of forming micro-infarctions.

Furthermore, we discovered the role of fasting blood glucose on administration in the development of R-DWIL. Glucose metabolism disorders may be pre-existed but occult, which may be aggravated due to the ICH ictus. Therefore, as to our current study, past history of diabetes appeared having no correlation with R-DWIL occurrence. Yet, we should be cautious that hyperglycemia might be a transient neuroendocrine change happened during ICH injury. One assumption is that hyperglycemia is an indirect marker of ICH-associated stress, reflecting neurological severity of ICH (22). Under stress, the hormones cortisol and catecholamines are elevated, causing small cerebral arteries vasospasm, resulting in hyperglycemia and small infarcts simultaneously.

Koga et al. found that in ICH patients with hyperglycemia, the disruption of blood–brain barrier (BBB) was distinctly induced, not only in the perihematomal region but also in the more distant structures. Meanwhile, free radicals, superoxide, and inflammatory cytokines such as tumor necrosis factor-α and interleukin-1 were increasing substantively (23). A ruptured BBB was permeable to large molecules and facilitates the invasion of blood components and inflammatory cytokines into parenchyma. That ensuing inflammation and oxidation reaction brought about increased risk of cytotoxic edema (24). Since the imaging appearance of cytotoxic edema in both DWI and ADC are similar to R-DWIL’s, we here suggest the possibility that the R-DWIL implicates more severe cytotoxic cellular injuries. In univariate analyses, we found that ICH patients with ventricular extension were more likely to have R-DWIL. Previous study observed elevating cerebrospinal fluid inflammation profile after intraventricular hemorrhage, which support the occurrence of R-DWIL may be related with severe regional inflammation and BBB disruption (25).

However, whether R-DWILs were related to the cardiac embolism or *in situ* small vessel occlusion due to the vascular autoregulation impairment, or neurotoxic reaction with abominable cellular environment, is still obscure. To determine the origin of R-DWIL, more sensitive monitor or techniques are required.

This study had several limitations. The time interval of MRI scan ranged from 1 to 28 days after ICH onset in our study, adding to the heterogeneity of the data. Since we did not perform sequential DWI in single patient, there is a possibility that some R-DWIL may present in the early stage had faded by the time of the MRI scan, or some R-DWIL may occur after MRI scan. Thus, a consequential observation of patients may reflect the R-DWIL evolution better. Besides, patients were excluded from our study if clear MRI images were unavailable. This may lead to bias, since patients who had more severe hemorrhage and impaired consciousness are intend to be ruled out. Of these analyzed patients, the median GCS was 15, indicating relative mild illness. In sum, prospective large-scaled cohort with dynamic observation on R-DWIL evolution and long-term follow-up are urgent in our further exploration.

## Conclusion

Remote DWI lesions are common in patients with acute spontaneous ICH. Our work showed that higher blood fasting glucose and high-grade WMH are strongly associated with R-DWIL formation.

## Ethics Statement

This study was carried out in accordance with the recommendations of the Institutional Human Research Ethics Committee of the Second Affiliated Hospital of Zhejiang University with written informed consent from all subjects. All subjects gave written informed consent in accordance with the Declaration of Helsinki. The protocol was approved by the Institutional Human Research Ethics Committee of the Second Affiliated Hospital of Zhejiang University.

## Author Contributions

X-hY brought up the main idea, analyzed data, and wrote the manuscript. TG collected part of the data, searched for literatures, and gave some helpful suggestions. X-hX found some useful papers and provided helpful input on the theme. J-sC helped to read the images. J-wL collected part of the data. K-mL, X-zY, and S-jS offered available suggestions to write the manuscript. L-sT read part of the images, revised the manuscript, and polished the language. FG and L-sT supervised and offered guidance to all the authors and revised the whole framework.

## Conflict of Interest Statement

The authors declare that the research was conducted in the absence of any commercial or financial relationships that could be construed as a potential conflict of interest. The reviewer WK and handling Editor declared their shared affiliation.
